# Clinical trial simulations in pulmonary fibrosis: patient-focused insights and adaptations

**DOI:** 10.1183/23120541.00602-2022

**Published:** 2023-05-30

**Authors:** Steve Jones, Maxine Flewett, Ron Flewett, Sharon Lee, Bill Vick, Milla Thompson, Sabine Pinnetti, Donald F. Zoz, Anna-Maria Hoffmann-Vold, Michael Kreuter, Toby M. Maher

**Affiliations:** 1 The European Pulmonary Fibrosis Federation, Overijse, Belgium; 2 Action for Pulmonary Fibrosis, Peterborough, UK; 3 Pulmonary Foundation Trust, Lichfield, UK; 4 Canadian Pulmonary Fibrosis Foundation, Markham, ON, Canada; 5 PF Warriors, Plano, TX, USA; 6 Boehringer Ingelheim Finland Ky, Helsinki, Finland; 7 Boehringer Ingelheim GmbH & Co. KG, Biberach an der Riss, Germany; 8 Boehringer Ingelheim Pharmaceuticals Inc., Ridgefield, CT, USA; 9 Department of Rheumatology, Oslo University Hospital, Oslo, Norway; 10 Department of Rheumatology, University Hospital Zurich, Zurich, Switzerland; 11 Center for Pulmonary Medicine, Department of Pneumology, Mainz University Medical Center and Department of Pulmonary, Critical Care and Sleep Medicine, Marienhaus Clinic Mainz, Mainz, Germany; 12 National Heart and Lung Institute, Imperial College London, London, UK; 13 Keck Medicine of USC, Los Angeles, CA, USA

## Abstract

**Background:**

Patient recruitment and retention are a challenge when conducting clinical trials in patients with pulmonary fibrosis, including idiopathic pulmonary fibrosis and other interstitial lung diseases. This study aimed to understand and address the barriers associated with trial participation for these populations.

**Methods:**

Nine patients, nine caregivers and three healthcare professionals participated in virtual simulations of planned phase III trials. During the simulations, participants received information about the trials and either tested a home spirometry device or watched a home spirometry demonstration, before providing their insights in debriefs. The findings were interpreted in advisory boards with representatives from patient organisations and expert investigators.

**Results:**

Regarding barriers to participation, patient fatigue and breathlessness were emphasised as posing challenges for travel, visit length and completion of onsite assessments. Lack of information, support and appreciation were also identified as factors that may exacerbate anxiety and negatively affect participant retention rates. Feedback on the home spirometry was mixed, with participants appreciating being able to complete the test at home but worrying about device handling. Based on the insights gained, patient-friendly adaptations were made to the trial protocol and conduct, including remote assessment of patient-reported outcomes, increased visit flexibility, travel support services, patient and caregiver information campaigns, and training of investigators on patients’ needs.

**Conclusions:**

Participants identified important barriers to participation, which led to patient-friendly changes being made to the planned trials. As a result, participation in the planned trials should be less burdensome, with improved recruitment and retention rates, and ultimately, improved data quality.

## Abstract 2

**Insights were harnessed through simulations of planned trials in patients with pulmonary fibrosis, caregivers and healthcare professionals. Findings were analysed in patient and expert advisory boards, and patient-friendly changes to the trials were made.** https://bit.ly/3z5PIFH

## Introduction

Patients and their caregivers are experts in the day-to-day challenges of living with and managing their disease. They are also among the beneficiaries of clinical trial outcomes, but their knowledge and experiences have been both underappreciated and underutilised in the design and conduct of clinical trials. Although their insights continue to represent a largely untapped resource, there is a growing appreciation of the value of placing them at the centre of clinical development programmes [1–4].

This article describes patient, caregiver, healthcare professional (HCP) and expert involvement in the design and planning of two phase III clinical trials: one in patients with idiopathic pulmonary fibrosis (IPF; ClinicalTrials.gov: NCT05321069) and the other in patients with progressive pulmonary fibrosis (PPF; ClinicalTrials.gov: NCT05321082). IPF and PPF are interstitial lung diseases (ILDs) characterised by pulmonary fibrosis, reduced quality of life and increased mortality [5–11]. There remains an unmet need for additional treatments for these populations, who also experience delayed referrals and misdiagnoses [12–16].

As in other rare diseases, clinical trials in pulmonary fibrosis are at risk of slow participant recruitment and poor retention [17, 18], which may delay the delivery of results, inflate costs, bias findings and limit trial outcomes [19]. Although the hope for new and improved treatments now and in the future may motivate patients to participate in trials, trial procedures and follow-ups represent a considerable burden, particularly for patients with fast-progressing and debilitating disease. Understanding patient perceptions of barriers to participation is vital to reduce the burden for participants as well as to ensure that trial outcomes are relevant to them. Despite this need, there have been no prior studies on patients’ perspectives on and expectations for clinical trials in pulmonary fibrosis.

To understand and address barriers to trial participation, we conducted simulations of planned trials with patients with pulmonary fibrosis, caregivers and HCPs. We also held advisory boards with members of patient organisations (POs) and experts. The overarching objective was to reduce the burden of trial participation, with a view to also improving data quality and future recruitment and retention rates.

## Methods

### Study design

The study was conducted virtually during March–May 2022 with patients, caregivers and HCPs from the USA, Spain and Japan.

**FIGURE 1:**
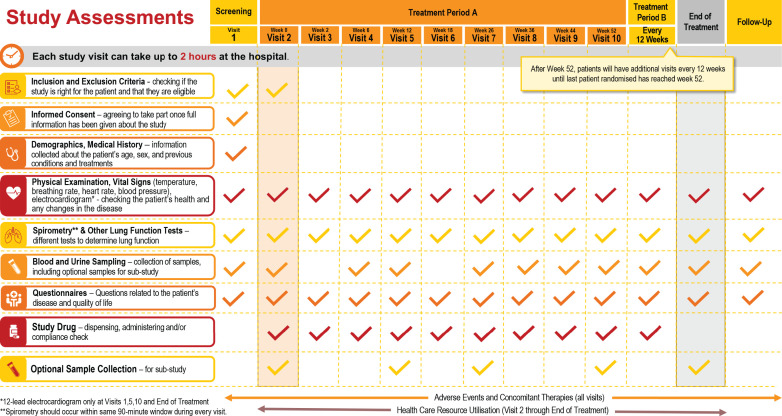
Overview of visit and assessment schedule for the planned trials as shown to participants.

**FIGURE 2:**
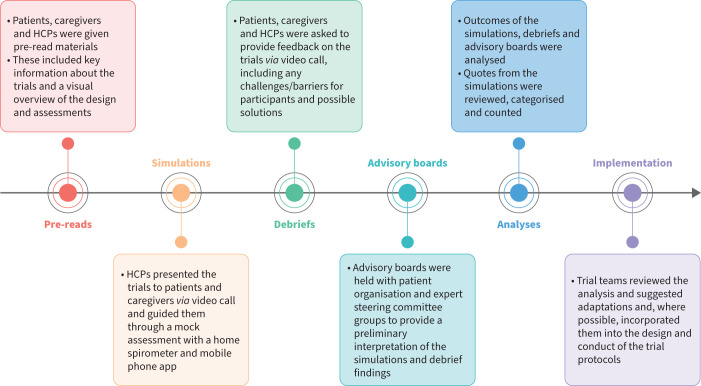
Overview of study design and methods. HCP: healthcare professional.

Participants completed a survey on their backgrounds and reviewed materials related to the planned trials, including the schedule of visits and assessments (figure 1). They then took part in trial simulations, during which participants received further information and either tested a home spirometry device or watched a home spirometry demonstration, before providing their insights in debriefs. The findings were interpreted in advisory boards with PO representatives and experts, who made recommendations for patient-friendly adaptations to the trials. Study teams reviewed the recommendations and, where possible, incorporated them into the design and conduct of the trials (figure 2).

Details on two initial PO advisory boards that were held to inform the study are provided in the supplementary material, along with details on the surveys and materials.

### Participants

Participants were recruited from an opt-in market research panel by a third-party agency (HealthiVibe; https://healthivibe.com) with no involvement of the study sponsor. Written informed consent was obtained from all participants prior to their enrolment. All procedures were conducted in accordance with the Declaration of Helsinki. The study was reviewed by Sterling Institutional Review Board (9745; www.sterlingirb.com) and received a Category 2 exemption from full institutional review in accordance with the regulations of the US Department of Health and Human Services [20].

Patients were eligible for participation if they were aged 40–65 years, had a diagnosis of fibrosing ILD (IPF or pulmonary fibrosis/other ILD) and were accompanied by a caregiver who agreed to participate. HCPs were eligible if they were experienced in trials and in managing patients with ILD. For each of the countries, the target was to recruit three pairs of patients and caregivers and at least one HCP.

### Trial simulations

Trial simulations were led by HCPs, conducted with pairs of patients and caregivers in their native language, and split into two parts.

During the first part, HCPs explained the rationale for and design of the planned trials. Randomisation and the possibility of receiving a placebo were discussed, along with the schedule of assessments and trial duration. Sample questions from patient-reported outcomes (PROs) were also shown. The first part of the simulations lasted ∼60 min.

During the second part, HCPs asked patients and caregivers about their familiarity with spirometry and oximetry, before explaining the measures they provide, the rationale behind their inclusion and their importance in the context of pulmonary trials. In the USA and Spain, this was followed by a mock assessment with a home spirometry device and mobile phone app (iSpiro; https://clario.com/solutions/respiratory), with participants being trained either *via* live virtual instruction (USA) or by viewing an instructional video (Spain). For logistical reasons, participants in Japan viewed the instructional video without trialling the device. The second part of the simulations lasted ∼45 min.

The simulations were recorded and transcribed. Japanese and Spanish transcripts were translated into English by professional translators (Acolad Life Sciences, New Rochelle, NY, USA) with native language proficiency, ensuring accurate and precise language. A certificate of translation was provided with each translated transcript.

### Debrief interviews

Within 6 days of the simulations, participants took part in separate virtual debrief interviews with an independent moderator, contracted by the study sponsor.

Moderators followed semistructured debrief interview guides, with questions relating to the following focus areas: trial design, trial assessments, trial visit schedule and duration, and participant support. During the debriefs, participants additionally rated their agreement with statements relating to the trial on a 5-point Likert scale. Statements were framed to ask how ratings might change if possible barriers to participation were addressed. Example questions and statements are provided in the supplementary material. Debriefs were conducted in the native language of moderators and participants, and lasted ∼100 min. Simultaneous interpreters (CorEvitas, Waltham, MA, USA) provided real-time English language translation for debriefs conducted in Japanese and Spanish. This industry standardised process ensured seamless communication without interfering with the natural flow of conversation for moderators and participants. Debriefs were recorded and transcribed.

### Advisory boards

Advisory boards were first conducted with a PO advisory group and then with experts. The purpose of the advisory boards was to provide a preliminary analysis of the insights from the simulations and debriefs, as well as to make suggestions for patient-focused adaptations to the planned trials. For identified barriers to participation, recommendations for possible solutions were generated.

### Analysis

Demographic characteristics, survey responses and participant ratings of statements were summarised using descriptive statistics. Based on the debrief transcripts, a thematic analysis was conducted by raters with experience in qualitative research. The semistructured format of the debrief interviews ensured coverage of predetermined focus areas while also allowing for the emergence of novel themes and insights. One rater first reviewed the translated transcripts and identified key quotes as those that would be important to patients, the sites/investigators and/or the trial team. They then grouped the key quotes by predetermined focus area and generated a simple coding framework in which quotes were categorised according to whether they included barriers/challenges for participants, positive feedback and/or suggestions for adaptations. A second rater independently reviewed the framework and applied it to the quotes. Agreement was high, but where minor discrepancies arose, these were resolved through discussion. For each focus area, quote counts were presented by category along with a representative selection of anonymised key quotes. To identify and refine novel themes and insights, the anonymised key quotes were further analysed and discussed during the advisory boards with the PO advisory group and experts.

## Results

### Participant and advisory board characteristics

Nine pairs of patients and caregivers participated in trial simulations and debriefs. Of these, four were from the USA, three from Spain and two from Japan. Two patients had a self-reported diagnosis of IPF and seven of other ILDs. Six caregivers were spouses or partners of patients and three were adult children. None of the patients had prior experience of participating in an ILD clinical study, but one caregiver had previously supported a patient in a study. Further demographic characteristics are presented in tables 1 and 2.

**TABLE 1:** Characteristics of patients who participated in trial simulations

**Patients**	9 (100.0)
**Male**	3 (33.3)
**Age**	
40‒49 years	3 (33.3)
50‒59 years	1 (11.1)
60‒65 years	5 (55.6)
**Country**	
USA	4 (44.4)
Spain	3 (33.3)
Japan	2 (22.2)
**Type of ILD**	
Idiopathic pulmonary fibrosis	2 (22.2)
Pulmonary fibrosis/other ILD	7 (77.8)
**Age at diagnosis**	
20–39 years	2 (22.2)
40–59 years	6 (66.7)
<60 years	1 (11.1)
**Time living with disease**	
<10 years	6 (66.6)
>10–19 years	3 (33.3)
**Symptoms experienced**	
Breathlessness	8 (88.9)
Cough	7 (77.8)
Depression/anxiety	1 (11.1)
Fatigue	8 (88.9)
Weakness	4 (44.4)
Weight loss	1 (11.1)
Urinary incontinence	1 (11.1)
Decreased mobility	2 (22.2)
**Use of supplemental oxygen**	
Yes, occasionally/rarely	5 (55.6)
No	4 (44.4)
**History of, or on waiting list for, lung transplantation**	
Yes	0 (0.0)
No	9 (100.0)
**Prior experience in a fibrosing ILD clinical study**	
Yes	0 (0.0)
No	9 (100.0)
**Current participation in a patient organisation or support group**	
Yes	4 (44.4)
No	5 (55.6)

Data are presented as n (%). ILD: interstitial lung disease.

**TABLE 2:** Characteristics of caregivers who participated in trial simulations

**Caregivers**	9 (100.0)
**Male**	3 (33.3)
**Age**	
18‒29 years	1 (11.1)
30‒39 years	1 (11.1)
40‒49 years	1 (11.1)
50‒59 years	2 (22.2)
60‒69 years	3 (33.3)
70‒79 years	1 (11.1)
**Country**	
USA	4 (44.4)
Spain	3 (33.3)
Japan	2 (22.2)
**Relationship to patient**	
Spouse or partner	6 (66.7)
Adult child	3 (33.3)
**Residing with patient**	
Yes	7 (77.8)
No	2 (22.2)
**Support provided to patient**	
Housekeeping	8 (88.9)
Meal preparation	7 (77.8)
Medication reminders and administration	6 (66.7)
Transportation assistance	5 (55.6)
Scheduling/reminding of doctors’ appointments	4 (44.4)
Personal care such as dressing and bathing	1 (11.1)
Other (unspecified)	1 (11.1)
**Time spent supporting patient per week**	
<20 h	4 (44.4)
>20 h	5 (55.6)
**Prior experience supporting a patient in a clinical study**	
Yes	1 (11.1)
No	8 (88.9)
**Current participation in a caregiver organisation or support group**	
Yes	1 (11.1)
No	8 (88.9)

Data are presented as n (%).

From each of the countries, one HCP led the simulations and took part in debriefs. In Spain and the USA, both HCPs were pulmonologists, whereas in Japan the HCP was a respiratory therapist.

Two advisory boards were held with the PO advisory group, which was comprised of four patient representatives and one caregiver. A further advisory board was held with three experts who were investigators on the trial steering committees.

### Insights on the planned trials

**FIGURE 3:**
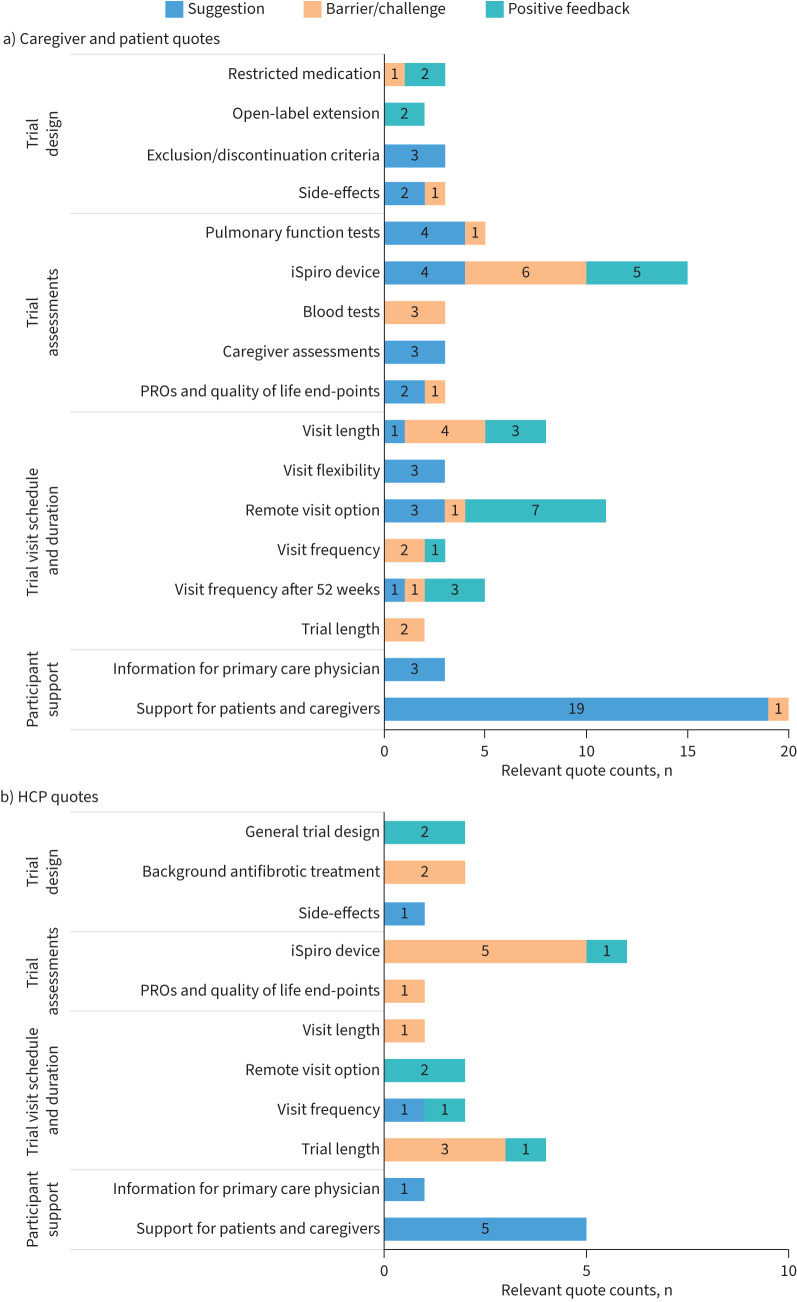
Relevant quote counts by focus area and category: a) caregiver and patient quotes, and b) healthcare professional (HCP) quotes. PRO: patient-reported outcome.

Novel themes, insights and suggestions from the simulations and advisory boards are summarised by focus area in the following subsections. For each focus area, relevant quote counts from the simulations and debriefs are presented by category in figure 3 and key representative quotes are presented in the supplementary material.

### Trial design: general impressions

**FIGURE 4:**
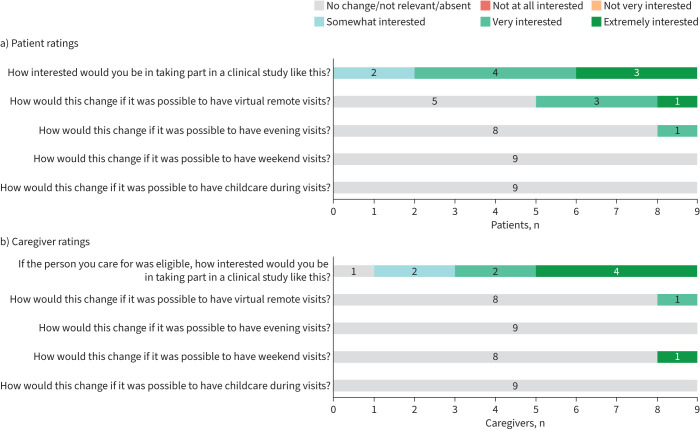
Participant ratings: a) patient ratings and b) caregiver ratings.

Regarding the planned trials, almost all patients and caregivers indicated that they were interested in taking part, with seven patients (77.8%) and six caregivers (66.7%) indicating that they were very or extremely interested (figure 4).

Most patients and caregivers agreed that it is important for caregivers to be involved in the decision to participate. The importance of involving caregivers was reiterated in advisory boards with the PO group, who also noted that caregivers may be more cautious than patients. Patients emphasised that a sense of altruism and helping others were reasons to participate.

Two patients provided insights that were relevant to the exclusion criteria, with both noting the lack of services and opportunities available for patients with pulmonary fibrosis caused by coronavirus disease 2019 (COVID-19).

HCPs were generally positive about the design of the planned trials, including the open-label extension, remarking that it was “appropriate” and “on a par with” other trials in these populations. HCPs also noted that as patients would be allowed to continue receiving standard of care, randomisation to placebo should not pose major challenges.

Two patients requested more information about side-effects of the trial medication. While one HCP noted that further information may improve participant retention, another remarked that it would be important to establish the side-effect profile in participants receiving background antifibrotic treatment alongside the trial medication. Concerns around side-effects were echoed in advisory boards with the PO and expert groups, who reiterated that further information should be provided to trial participants and caregivers.

### Trial assessments

Regarding the trial assessments, patients and caregivers accepted the need for, and proposed frequency of, in-person pulmonary function testing. However, one patient emphasised that they found pulmonary function tests “challenging” and another that they felt pressure to “get it right”. Similar concerns were raised in advisory boards with the PO and expert groups, who noted that spirometry can cause anxiety.

Based on the simulations, patients, caregivers and HCPs all expressed mixed feelings about home spirometry and the iSpiro device. Participants were generally very enthusiastic about the prospect of being able to complete the test at home, with one patient emphasising that it was “fantastic” and “miraculous” to not have to go to the hospital. However, participants also voiced concerns about handling the device. This was particularly relevant for those participants from the USA and Spain who were unable to obtain a complete measure, and who therefore suggested that further support be provided from a technician during onboarding. The patient and caregiver from Japan voiced additional concerns around device cleanliness and maintenance.

In discussions about the app used to collate spirometry data, all three HCPs expressed general reservations about new technologies for remote data collection, remarking that as these populations tend to be of an older demographic, new technologies need to be user friendly and easy to handle. The need for real-time one-to-one support during onboarding was also emphasised in advisory boards with the PO and expert groups.

Patients and caregivers reported finding PROs challenging to complete during in-person study visits due to not feeling relaxed or feeling time pressured. As such, most indicated that they would prefer to complete PROs at home. One HCP remarked that due to the sensitive nature of some PROs, discussing them in person can be “scary” and requires a degree of trust. One patient noted that while lung function outcomes were most important to pulmonologists, quality of life outcomes, particularly those involving activities of daily living, were most important to them.

Three caregivers indicated that it would be useful to include caregiver measures (*e.g.* on patient symptoms) alongside PROs, perceiving them as an important source of additional information. The expert group agreed but noted that future studies are needed to assess whether such measures predict future outcomes.

### Trial visit schedule and duration

Patients, caregivers and HCPs generally considered the trial length, visit length and visit frequency to be acceptable, although several participants indicated that the overall commitment represented a burden. While 2-h visits were generally perceived to be manageable, one patient noted that chronic fatigue is common among patients with ILDs; another emphasised that due to this, 2 h would be at “the outer limit” of what they could manage, particularly if travel time would be required. The PO advisory group similarly remarked that some trial participants may find the schedule of visits to be onerous. They also emphasised that the ability to cope with trial procedures may vary between patients.

When asked whether evening hours, weekend hours or childcare would affect their interest in participating, most patients and caregivers indicated that these were not relevant due to their life stage, with most being retired with adult children. One caregiver also remarked that patient energy levels tend to peak in the morning and decline in the afternoon and evening.

While five patients and eight caregivers indicated that virtual visits would not affect their interest in participating (figure 4), many were very positive about having the option (figure 3), particularly in relation to COVID-19 or when feeling unwell (see supplementary material). One caregiver emphasised that they felt that in-person visits were important for patient monitoring and one patient reported preferring in-person visits due to their difficulties with technology.

### Participant support

Regarding participant reimbursement and compensation, including for caregivers, participants were positive, with one patient noting that caregivers “have costs” and “skin in the game”. The PO and expert advisory groups similarly emphasised that caregivers play a crucial role in supporting and motivating patients throughout trials, with the PO group suggesting that they should be compensated for any loss of income associated with attending visits. The need to make caregivers feel visible and valued, as well as to reimburse them for costs incurred while supporting trial participants, was highlighted, with caregiver support identified as one of the top priorities by the PO group. The PO group additionally noted that trial participants do not always feel supported or appreciated.

When asked about how else participants might be supported, patients and caregivers suggested that it would be good to have a schedule of visits and to be reminded of them, with one patient remarking that this would make them feel “more safe”.

Most patients and caregivers also indicated that site accessibility can be challenging, particularly for wheelchair users and patients receiving oxygen therapy. In this context, one patient reiterated concerns around fatigue and another around breathlessness. Similar concerns were voiced by the PO group, who emphasised that using public transport can be difficult for these populations. To ease the burden, the PO group suggested that travel assistance be provided.

The importance of a single point of contact was emphasised by patients and the PO group. Caregivers and HCPs also highlighted the need for moral and psychological support, with one HCP reiterating that respiratory patients often have anxiety and another emphasising the importance of listening to their concerns. One caregiver noted that peer groups can be “really helpful” to “share experiences”. Another remarked that it would be good for participants to feel connected to the trial “beyond just a medical fact” and for it to “personally and psychologically give [them] something”.

When asked what information should be shared with trial participants’ primary care physician, patients and HCPs suggested that it would be important to share test results. They also emphasised that results should be shared with participants’ pulmonologists if they are not among the investigators.

### Adaptations to planned trials

**FIGURE 5:**
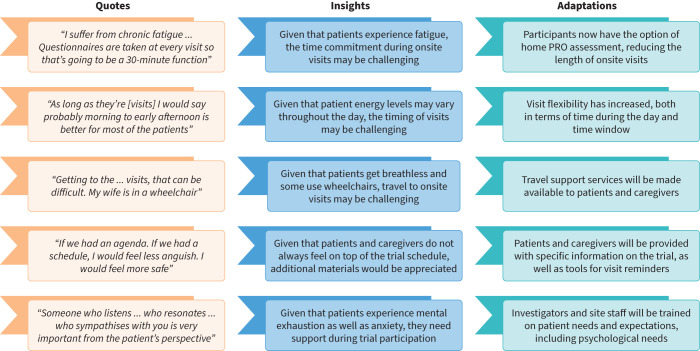
Summary of key quotes, insights and adaptations. PRO: patient-reported outcome.

Based on these novel themes and insights, several changes to the trial protocol and conduct were made, including around home assessments, visit flexibility, travel support and information campaigns. The most important changes are described in table 3 and summarised in figure 5.

**TABLE 3:** Description of the most important changes to the trial protocol and conduct

**Focus area**	**Adaptations to trial protocol and conduct**
**General trial design**	
Exclusion criteria	Having been excluded from prior trials, patients with pulmonary fibrosis caused by COVID-19 are now able to participate if their infection occurred at least 12 months before screening
**Trial assessments**	
Patient-reported outcomes	Participants have the option of completing PROs at home (*via* paper or electronically *via* a smartphone app), reducing the length of onsite visits
**Trial visit schedule and duration**	
Visit flexibility	Extra flexibility has been allowed for visits, both in terms of time window (±14 days in later parts of the study) and time during the day
**Participant support**	
Site accessibility	Travel support services^#^ will be offered to participants and caregivers
Visit guide and reminders	Participants and caregivers will be provided with tools for visit reminders
Caregiver reimbursement	Where local regulations allow, caregivers will be reimbursed for expenses associated with supporting trial participants
Information campaigns	Regular participant and caregiver newsletters/information campaigns will be available onsite and on trial-specific websites
Investigator training	Investigators will be trained on patient needs and expectations, including psychological needs
Peer support	The trial sponsor is connecting with patient organisations to support trial-specific patient and caregiver ambassador programmes
Trial navigators	The trial sponsor is facilitating the establishment of trial navigators who will serve as a single point of contact and onsite support

^#^: where possible, travel support services will include pick-ups at and drop-offs at sites (the implementation will vary across sites depending on local regulations and the ability of sites to organise transport directly or *via* a third party). PRO: patient-reported outcome.

## Discussion

Our study harnessed patient-focused insights through simulations of planned phase III trials with patients with pulmonary fibrosis, caregivers and HCPs, and advisory boards with PO members and experts. It also resulted in patient-friendly adaptations to the design and conduct of the planned trials.

Although similar work has been conducted in other therapy areas (*e.g.* lupus and systemic sclerosis-associated ILD [21, 22]), our study is the first to provide the patient and caregiver perspective on clinical trials in IPF and other fibrosing ILDs. Among the key insights, patient fatigue and breathlessness were emphasised as posing challenges for travel, visit length and completion of onsite assessments. Lack of information, support and appreciation were also identified as factors that may exacerbate anxiety and negatively affect participant motivation. Feedback on the home spirometry was mixed, with participants appreciating the prospect of completing the test at home but worrying about device handling. The insights gained led to some important changes to the planned trials, including the option of home PRO assessment, increased visit flexibility, travel support services, patient and caregiver information campaigns, and training of investigators on patients’ needs.

Our findings provide a framework for how to involve patients and their caregivers in clinical research, and have implications not only for the planned trials, but also for future trials in patients with ILDs. Efforts to understand and incorporate the patient perspective are likely to improve the feasibility and quality of trials as well as their relevance to patients, who deserve to feel that their time sacrifice and contribution are valued and worthwhile. In recognition of this, more systematic patient involvement is increasingly becoming an expectation among regulators [23, 24]. In the UK, for example, the Medicines and Healthcare Products Regulatory Agency is working towards integrating patient involvement into the approval process for medicines [25, 26]. Despite this progress, there remains a lack of guidance around caregiver involvement in trials, including on reimbursement for the costs they incur while supporting participants.

Our study is, however, not without limitation. Due to timelines and to facilitate recruitment, patients were included based on their self-reported diagnosis, and those over the age of 65 years were excluded. Unlike in the planned trials, formal criteria for progressive fibrosis were not assessed. This may have limited the generalisability of our findings to younger patients with less progressive disease, while the planned trials will enrol older patients with progressive disease. Also, most patients and caregivers were interested in trial participation from the outset, which made a potential increase in their interest in participating difficult to quantify. Moreover, the participant sample was small, which prevented meaningful examination of possible cultural differences.

Central to our study is our inclusion of patients and caregivers, as well as HCPs and experts. Our findings underscore the need for early and extensive involvement of all parties in trial design and planning. As a result of our integration of perspectives, we believe that participation in the planned trials will be less burdensome, which should lead to improved recruitment of a wider population and facilitate long-term patient contributions. In turn, this should improve data quality and ultimately, we hope, contribute to the faster provision of new medicines to patients.

## Supplementary material

10.1183/23120541.00602-2022.Supp1 **Please note:** supplementary material is not edited by the Editorial Office, and is uploaded as it has been supplied by the author.
Supplementary material 00602-2022.SUPPLEMENT
