# Supplementary material for: Clinical trial simulations in pulmonary fibrosis: patient-focused insights and adaptations
Source: ERJ Open Res. 2023 May 30;9(3):00602-2022. doi: 10.1183/23120541.00602-2022 (PMC10227627; doi:10.1183/23120541.00602-2022)
Supplement: Supplementary file 1 [file 00602-2022.SUPPLEMENT.pdf]

Supplementary material

Clinical trial simulations in pulmonary fibrosis: patient-focused insights and adaptations

Steve Jones, Maxine Flewett, Ron Flewett, Sharon Lee, Bill Vick, Milla Thompson, Sabine Pinnetti, Donald F. Zoz, Anna-Maria Hoffmann-Vold, Michael Kreuter, Toby M. Maher

Contents

Section 1: Plain language summary 2
Section 2: Patient organisations involved in advisory boards 3
Section 3: Surveys and pre-read materials 5
Section 5: Examples of debrief questions and rating statements 13
Section 6: Key representative quotes 15

Section 1: Plain language summary

Clinical trials are important for the development of new medicines. For clinical trials to work, enough patients must take part. This can be hard in rare lung diseases. Understanding what patients find difficult in clinical trials could help to improve how they are run.

This simulation study aimed to understand and find ways to improve the patient experience of taking part in clinical trials. During the simulation, patients with lung diseases and their carers received information about two planned trials. They were then asked to provide feedback. Their feedback was discussed with members of patient organisations and experts, who made suggestions for how to make the planned trials easier for those taking part.

Patients and carers said that breathing problems and exhaustion could make it difficult for patients taking part in the trials to travel to the hospital for tests. They also indicated that those taking part should be given more information and support. Based on this feedback, patient-friendly changes were made to the planned trials. The most important changes include:

- Patients will be able to complete some tests at home
- There will be more flexibility around the timing of hospital tests
- Patients and carers will be supported with travel to the hospital for the trial
- More information will be provided to patients and carers
- Trial staff will be trained on patients’ needs

These changes should improve the trial experience for those taking part, which, in turn, should improve the quality of the trial.

Section 2: Patient organisations involved in advisory boards

Initial advisory board

In November 2021, an initial virtual advisory board was held with patient representatives and caregivers from 18 patient organisations (POs) from across the world, including:

- Action for Pulmonary Fibrosis (UK)
- Association Fibroses Pulmonaires (France)
- Canadian Pulmonary Fibrosis Foundation (Canada)
- Deutsche Sarkoidose-Vereinigung e.V. (Germany)
- European Pulmonary Fibrosis Federation (EU)
- Foundation for Sarcoidosis Research (US)
- Idiopathic Pulmonary Fibrosis Association Bulgaria (Bulgaria)
- Irish Lung Fibrosis Association (Ireland)
- PF Warriors (US)
- Pulmonary Fibrosis Foundation (US)
- Pulmonary Fibrosis Trust (UK)
- Sjögren's Foundation (US).

During this meeting, an overview of the simulation study was presented to gauge patient interest.

Study advisory board group

To gain further insights on the proposed design, an additional advisory board was held in February 2022 with five representatives of the following POs who had agreed to be part of an advisory group for the simulation study:

- Canadian Pulmonary Fibrosis Foundation (Canada)
- European Pulmonary Fibrosis Federation (EU)
- PF Warriors (US)
- Pulmonary Fibrosis Trust (UK).

Section 3: Surveys and pre-read materials

Surveys

To obtain basic demographic information, patients and their caregivers completed a virtual survey. For patients, the survey included questions relating to their diagnosis and medical history, whereas for caregivers the survey instead included questions relating to their relationship to the patient. For both patients and caregivers, the survey included questions relating to their current participation in patient support groups, their prior experience with clinical trials, how patients should be informed about clinical trials, and whether it is important for caregivers to be involved in a patients' decision to participate in a clinical trial.

Pre-read materials

Patients, caregivers and HCPs received general information on clinical trials, as well as study-specific information to read in advance of the trial simulations. This included a succinct overview of the planned design of the Phase III trials, along with the visit schedule, assessments, permitted medications and what to expect during the trial simulations. The study sponsor was not mentioned in the pre-read materials, and participants remained blinded to the sponsor throughout the study. Example materials are provided below.

Section 4: Examples of questions and answers from the pre-read materials for patients and caregivers

Clinical Trial Simulation with Patients Diagnosed with Fibrosing Interstitial Lung Disease (ILD): Pre-Read for Patient / Caregiver Participant

DRAFT

Questions & Answers – About Clinical Trials in General

1. What is a clinical trial?

The development of a new medicine is a complex process that takes an average of 12-15 years.

The process starts with researchers studying diseases that lack satisfactory treatments. They try to understand how people with the disease differ from those who do not have the disease. Researchers also try to understand the processes that are changed or altered in people with the disease at the level of the cells and molecules inside the body. The next step is to try to find drugs that can fix these altered processes.

Before trying a possible new drug in humans, researchers conduct a lot of testing in living cells and animals to see if the drug works as expected, or causes harm.

After this initial (preclinical) testing has been completed, the researchers, together with health authorities and Ethics Committees, review the data and decide whether the drug can be tested in humans. Studies in humans are called clinical trials. The aim of clinical trials is to find out whether a drug works and is safe for humans, and can be used as a medicine for humans with that specific condition.

The most reliable evidence comes from clinical studies in which the participants are randomly assigned to receive either the new drug or a control (usually placebo or the treatment that is commonly used for that disease, called 'standard of care'), and they don't know in what group they are. It is important to have a control group that does not receive the potential new drug because otherwise it would not be possible to conclude if the new drug is helping people with the condition.

Observed benefits or risks may occur by chance or because of factors other than the drug: people in studies often receive better healthcare, and this may improve their disease, independent of the treatment. It is also important to randomly assign study participants to either treatment or control since doctors may assign (consciously or subconsciously) sicker people to the drug and healthier people to the control, which may lead to an over- or underestimation of the treatment effect. Patients and doctors should not know who takes the treatment or control because psychological factors, like hope that the treatment will work, can influence perceptions and evaluations of patients.

Clinical trials are conducted in **four phases**. If a potential new drug is successful in one phase, it moves to the next phase.

Phase I: In the first phase of clinical development, researchers want to find a safe dosing range of the new drug and understand how the new drug should be given and how it behaves in the human body. The new drug is given to a small number of people (healthy volunteers or people with the disease) who are closely monitored.

Phase II: In the next phase, researchers want to find out the most effective and safe dose. Phase II trials are bigger: they include up to several hundred people living with the disease being studied.

Phase III: Phase III trials aim to confirm how well the new drug works, called 'efficacy,' and the possible side effects, called 'safety,' in a large patient population over a longer period of time. To ensure that the efficacy and safety results are not observed by chance, a large number of patients from different countries have to be included (up to thousands).

Before the start of every trial, a protocol is developed. Clinical trial protocols are documents that describe every detail of how the trial will be conducted. They are developed with guidance from experts and health authorities. The protocol is reviewed and approved by health authorities and Ethics Committees before a trial begins. The protocol describes:

- Who and how many people should participate in the trial?
- Will there be a control group?
- Will people be allocated randomly to a treatment group?
- How long will it last?
- What outcomes will be tested?
- What assessments and examinations will be done?
- How will the new drug be given and at what dose?

All our clinical trials follow the guidelines developed by the International Conference on Harmonisation and the World Medical Association Declaration of Helsinki on the Ethical Principles for Medical Research Involving Human Subjects.

- Before patients participate in a trial, they provide informed consent.
- During a trial, the safety of all participants is closely monitored.
- Participants can leave the trial at any time.
- For some trials, all participants (including those previously taking control) are allowed to take the study drug after the trial has ended – even if it will be several more years until the drug is on the market.
- When the results are available, they are published in a timely and transparent manner.

After the Phase III results are available, all data collected during the trial are sent to the health authorities. The health authorities thoroughly examine all data. If they find that the benefits of the medicine outweigh the risks, they give their approval for the new medicine to be marketed.

When the medicine is finally available to patients, the researchers continuously monitor the safety of the medicine. Sometimes, more clinical trials are done to collect further information. **These are called Phase IV clinical trials.**

DRAFT

Questions & Answers – About This Clinical Trial

2. What is the purpose of the trial?

One Phase III trial for patients with idiopathic pulmonary fibrosis (IPF) and one Phase III trial for patients with another type of progressive fibrosing interstitial lung disease (PF-ILD), are planned. Except for the diagnosis of the patients participating in the trial, the design of both these trials will be identical.

The purpose of these trials is to further study the safety, tolerability, and efficacy of an oral PDE4 inhibitor especially looking at: (1) slowing of lung function worsening, (2) improvement in overall quality of life, and (3) reduced hospitalizations and mortality.

The aim of these trials is to:

- Find out whether there is any change in pulmonary function tests (PFTs) over time in participants taking the study drug. At the time of enrolment, participants may or may not be taking an antifibrotic medication. They will be able to continue taking this medicine with the study drug.
- Determine the effect of the study drug on the progression of disease, as well as hospitalization and mortality rates.

3. How many people have taken the study drug so far?

As of December 2021, 5 clinical studies have been completed. These studies were done with approximately 50 healthy volunteers and 150 patients with interstitial lung diseases (ILD).

4. What safety results are available so far for the study drug?

In the people who have received the study drug so far, most frequent side effects were related to gastrointestinal symptoms like diarrhea, which occurred 2-3 times more often under the study drug than with placebo. These symptoms are also known to occur when taking the marketed antifibrotic medications.

5. What efficacy results are available so far for the study drug?

A double-blind, placebo-controlled Phase II trial was conducted studying three aspects of the study drug: efficacy, safety and tolerability in patients diagnosed with IPF over 12 weeks. The trial results provided early clinical evidence that this study drug showed significant improvement in Forced Vital Capacity (FVC) decline in patients with IPF.

This was true when the study drug was both given alone or with currently available antifibrotic therapy as compared to placebo. Diffusing capacity is a measure of how well oxygen and carbon monoxide are transferred (diffused) between the lungs and the blood. This test is known as DLCO and can be a useful in monitoring treatment of lung diseases. Results on change from baseline in DLCO showed the positive effect of the study drug on FVC. It also showed favorable results with the study drug in both patient groups (with or without concurrent antifibrotic treatment).

DRAFT

6. What criteria do I have to meet to take part in the study?

Inclusion Criteria	Exclusion Criteria
• Males and females, > 18 years old<br>• Diagnosis of IPF or non-IPF progressive fibrosing ILD within the past 12 months<br>• HRCT scan from the last 12 months needs to be available for central assessments and confirmation of the diagnosis<br>• On stable therapy antifibrotic meds (nintedanib or pirfenidone) for at least 8 weeks prior to Visit 1, with no plans to discontinue this treatment. OR<br>• Have not taken antifibrotic meds (nintedanib or pirfenidone) for at least 8 weeks prior to Visit 1 (either naïve or discontinued), with no plans to (re-)start this treatment<br>• Specified FVC and hemoglobin percentages<br>• Both men and women of childbearing potential must use highly effective method of birth control[1]	• Acute IPF or ILD exacerbation within 4 months prior to screening, or relevant airways obstruction/pulmonary abnormalities<br>• Usage of antibiotics for a lower respiratory infection within 4 weeks prior to Visit 1<br>• Any serious, chronic condition including but not limited to: HIV, hepatitis, TB, malignancy, chronic liver disease, alcohol or substance abuse<br>• Any major surgery within 3 months of Visit 1<br>• Any documented active or suspected malignancy or history of malignancy 5 years prior to Visit 1<br>• Underlying chronic liver disease<br>• Myocardial infarction (MI), unstable angina or history of thrombotic event within 6 mos of Visit 1<br>• Severe hypertension within 3 mos of Visit 1<br>• Uncontrolled or unstable vasculitis within 8 weeks prior to Visit 1

[1] A woman is considered of childbearing potential (WOCBP), i.e., fertile, following menarche and until becoming post-menopausal unless permanently sterile. Permanent sterilization methods include hysterectomy, bilateral salpingectomy, and bilateral oophorectomy. Tubal ligation is NOT a method of permanent sterilization

7. Do I have to stop my current medications to take part in the study? If I have to stop my current medications to take part in the study, how long before the study start do I have to stop them?

Patients are allowed to use immunomodulatory medications at a stable dose at study entry according to the eligibility criteria.

During the treatment period, addition or changes in immunomodulatory treatment are allowed for the management of worsening of the underlying disease as medically indicated.

Antifibrotic medications (nintedanib and/or pirfenidone) are allowed, but with no changes in this status in the first 12 weeks after starting intake of the study drug.

Restricted Medications				
Medication	Prior to Study	Screening Period	Treatment Period	Follow-Up Period
**Potent CYP3A inhibitors**	Permitted	Not Permitted	Not Permitted	Permitted
**Nintedanib/Pirfenidone (AF GROUP)** Antifibrotic stable at Visit 1	Permitted	Permitted	Permitted	Permitted
**Nintedanib/Pirfenidone (AF GROUP)** Antifibrotic stable at Visit 1	Not Permitted	Not Permitted	Not Permitted (allowed after 12 weeks of study participation with respective worsening of symptoms)	Permitted
**PDE inhibitors**	Not Permitted	Not Permitted	Not Permitted	Not Permitted
**Prednisone>20mg/day or equivalent**	Not Permitted	Not Permitted	Not Permitted	Permitted

8. What outcomes will be measured during the study?

Determine whether there is:

- Reduction in lung function decline
- Improvement in overall quality of life
- Reduction of hospitalizations and mortality

9. Will there be financial compensation during the study?

You will be offered financial compensation covering your expenses.

10. What measures are in place at the study site to protect me from COVID-19?

Clinical study sites have established COVID-19-protective measurements as per local health policies and regulations.

11. What will happen if my ILD/IPF gets worse during the study?

Stopping treatment is the last action except in cases of severe side effects. However, situations where there is acute worsening, infection or aggravation might lead to a temporary treatment discontinuation. You may restart treatment after side effects decrease or your condition is stable again.

Another option would be the introduction of nintedanib or pirfenidone in patients who had not previously taken antifibrotic medications.

DRAFT

Any further treatments of the underlying disease are allowed and will be decided upon by your treating doctor.

You have the right to withdraw your consent at any time. If you decide to stop trial medication and participation, then the final assessments required at the end of treatment visit should be completed as soon as possible and the end of trial visit should be completed a minimum of 7 days after you stopped taking trial medications.

12. What will happen if my other diseases get worse during the study?

In case of a worsening of your other diseases, your treatment will be adjusted based on what you and your doctor think is best.

13. If the study drug works for me, how can I continue taking it after the study?

After the study, all participants will be offered the opportunity to receive the study drug for a longer period of time as part of an open-label study at no cost to you.

Table of Abbreviations

Abbreviation	Definition
ILD	Interstitial lung disease
IPF	Idiopathic pulmonary fibrosis
HRCT	High resolution computed tomography
FVC	Forced vital capacity
DLCO	Diffusing capacity for carbon monoxide
PFTs	Pulmonary function tests
PF-ILD	Pulmonary fribrosing intersititial lung disease
MI	Myocardial infarction

DRAFT

Overview of study design from the pre-read materials for patients, caregivers and HCPs

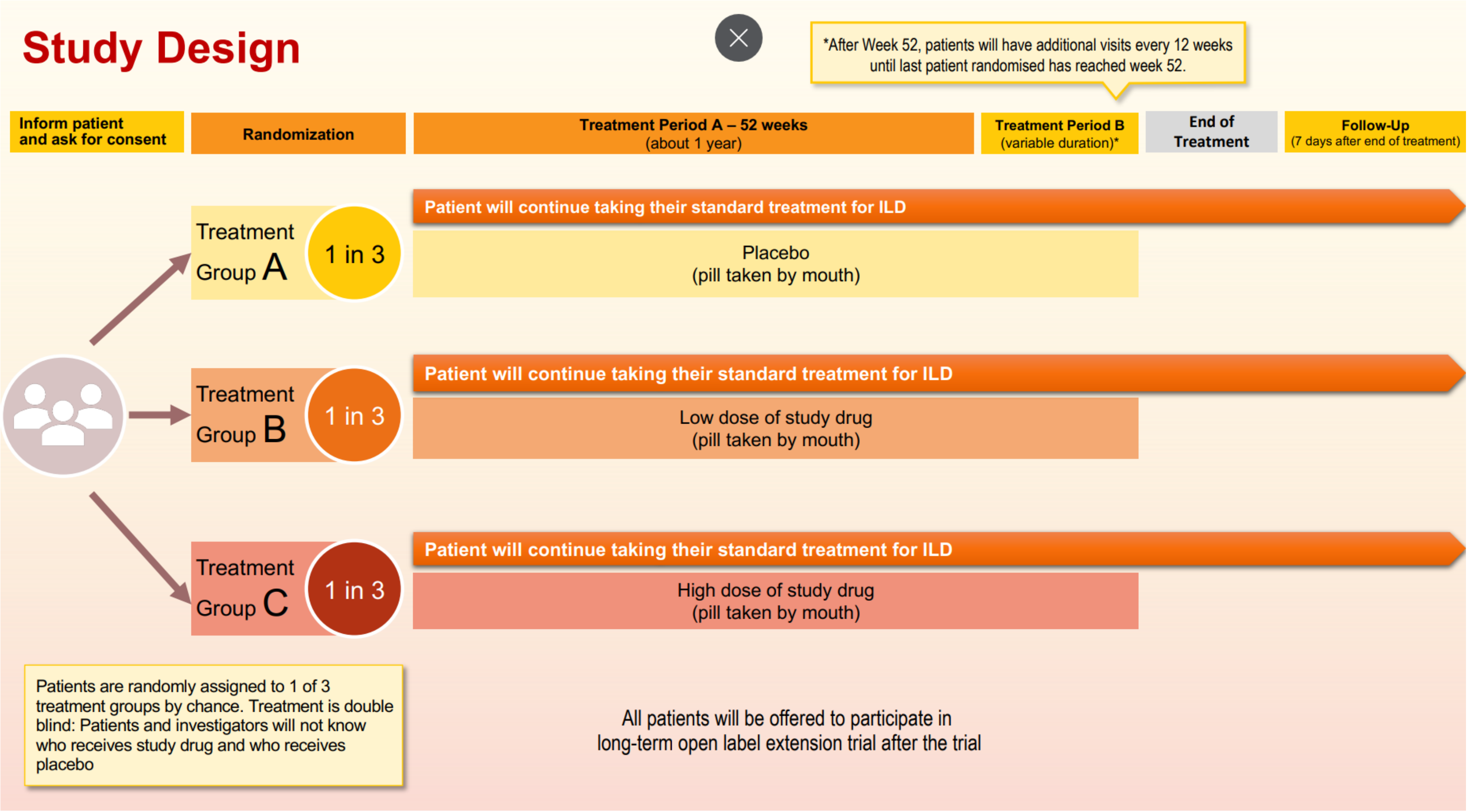

Section 5: Examples of debrief questions and rating statements

Examples of questions and rating statements from the patient debrief guide for moderators

Discussion guide	Time (indicative)
Let's start with some **general background information** about clinical trials.<br>• Have you ever considered participating, been asked to participate (or participated) in a clinical trial? Please explain.<br>o [IF YES] What was the experience like for you (e.g., length of time, requirements, overall experience, other comments)?<br>• Where would you go to find more information about clinical trials for your condition?<br>• What kind of information would you need/want to consider participating in a clinical trial?	5 min
Now, let's talk about your **general impressions of this trial.**<br>• Hypothetically, and assuming you are eligible, how interested would you be in taking part in a clinical study like this on a scale of 1 to 5, where 1 is "not at all interested" and 5 is "extremely interested"? Please explain.<br>• What did you think was good or what did you like about the planned study? Please explain. [PROBE]<br>• What concerns would you have about the study? Was the study design challenging in any way? Please explain. [FOR ALL CONCERNS RAISED: Ask how the concern could be resolved, what solution could help with the concern. Ask how it would impact their interest to join the study on the scale of 1-5 if the concern was resolved]<br>• What about your IPF/PF-ILD or other diseases would make it challenging for you to take part in a study like this? Please explain.<br>• If you were to participate in this kind of a study, what information would you want to know about the study drug?<br>• Do you have any suggestions to make it easier for patients to participate in this study?[FOR ALL SUGGESTIONS RAISED: Ask how it would impact their interest to join the study on the scale of 1-5 if the suggestion was implemented.]<br>• If you were thinking about joining a trial like this, what role or how important would the influence of others (family, physician, social media) be?<br>• What would be helpful in terms of communicating with your primary care physician (PCP) about participation in this kind of a study? What would you want the study to communicate to your PCP? In what form would you want this information communicated to your PCP (e.g., electronically, paper, patient portal, other)?	20 min

**Break**	10 min
The next topic is about the **study duration and visits**.<br>• What did you think of overall study duration of 52 weeks in Part A? Please explain.<br>• What did you think about the overall number, frequency and length of study visits? What were your reactions about the time between visits? Please explain.<br>• What would be a reasonable or realistic amount of time for your caregiver and you to be at a study site to complete visits before it becomes too burdensome for each of you? Please explain.<br>• You said at the beginning that your hypothetical interest in taking part in a study like this if you were eligible was a __ on a scale of 1 to 5, where 1 is "not at all interested" and 5 is "extremely interested". How would this interest change if study visits were every 16 weeks vs. every 8-12 weeks? [Note: first 3 visits have a short distance such as 2 and 4 weeks]<br>• Visits would also need to be conducted at the same time each time, i.e., within +/- 90 mins time window. How would this impact your interest and ability to participate? Please explain,<br>• You said at the beginning that your hypothetical interest in taking part in a study like this if you were eligible was a __ on a scale of 1 to 5, where 1 is "not at all interested" and 5 is "extremely interested".<br>○ How would this change if there was the possibility to have virtual or remote (video assisted) visits?<br>○ What if some of these visits were remote for instances when you felt unwell or were unable to travel to the site for other reasons, how might this impact your thoughts about the overall study length and time required? Please explain.<br>○ How would this change if there was the possibility to have visits on weekends? Please explain.<br>○ How would this change if there was the possibility to have visits late in the evening? Please explain.<br>○ How would this change if there was the possibility to have childcare during visits? Please explain.	15 min
The next topic is about **study assessments**.<br>• What concerns would you have about the study assessments? Please explain.<br>• What did you think about the number of blood samples that will be collected during the study?<br>• Patients will be asked whether they agree that additional samples to be collected would be pre-dose at baseline, and then again at 3 points in time (weeks 12, 26 and 52) may be stored and used to inform other research outside of the study to better understand the pathophysiology of IPF/PF-ILD. This so-called biobanking will be optional meaning you can decide whether the sample is collected, used and stored. For this, you give a specific consent. What do you think of this?<br>• What information should be provided to study participants to help with their decision whether to participate in biobanking?	15 min

Section 6: Key representative quotes

	Key patient and caregiver quotes	Key HCP quotes
**Trial design**		
General design, including open-label extension and restricted medication	*"I think it's good that, that patients can continue [trial medication] if they're receiving benefit"* (US patient, positive feedback)<br><br>*"I'm just worrying if something like that happened, and he needs to be hospitalized, and he needed to be on more prednisone than he normally is ... I see that it's not permitted"* (US caregiver, challenge/barrier)<br><br>*"No, the thing that worried me was, if he had to stop any of his meds that he was on now ... when I saw the list of meds, he wouldn't have to stop any of his regular meds and be taking placebo. I was OK with that even if he was just taking placebo"* (US caregiver, positive feedback)	*"Trial design is in line with any of the other big trials for IPF and PF-ILD, so appropriate trial design … no concerns about the study"* (US HCP, positive feedback)<br><br>*"No [placebo will not pose a challenge] because the patients are allowed to be on standard of care"* (US HCP, positive feedback)
Exclusion/ discontinuation criteria	*"We went to different hospitals, and they said it's a sequela of COVID and so we can't do anything with that. And they don't have any experience so we can't do anything"* (Japanese patient, suggestion)	-
Side effects of trial drug and use of background antifibrotics	*"Well the side effects, diarrhoea. I was wondering about that … that really stuck in my mind in terms of concerns"* (Japanese patient, challenge/barrier)<br><br>*"I would expect them to tell me potential adverse events … long term adverse events. I would like them to let me know about them … I always take into account whether it will be detrimental as well for me in terms of adverse events"* (Spanish patient, suggestion)<br><br>*"In principle, I'd like to know everything … I'd like to know if there has been secondary effects, I guess this has already been tried on humans. That's what I'd like to know"* (Spanish patient, suggestion)	*"We talked about … GI symptoms…. And then, there was a question about other symptoms. I couldn't really provide with a proper explanation. So in regards to all of the events that possibly could happen, I think that those things should be provided as an explanation properly and that they would prevent dropouts or help in preventing dropouts"* (Japanese HCP, suggestion)<br><br>*"I'm also concerned about it because I'm not used to treating a patient with two antifibrotics simultaneously, one on top of each other. Because liver toxicity is very frequent in patients with these type of treatments ... this will be one of the top questions"* (Spanish HCP, challenge/barrier)
**Trial assessments**		
Pulmonary function tests	*"I struggle with the pulmonary function tests just as my illness progresses the pulmonary function tests become more and more challenging … It'd be great if … somebody could ... come up with a better … a different PFT"* (US patient, challenge/barrier and suggestion)<br>*"But before you start, just say Jim, Mary, Tom, we have no idea where you're at. Just give us your best shot. You know what I mean? A little bit of a pep talk probably wouldn't hurt"* (US patient, suggestion)	-

iSpiro device and app	*"It was fantastic that you ... sent me the spirometer and the technology is incredible. The fact that I didn't have to go to a hospital … It's miraculous … So I don't know you guys make it pretty easy"* (US patient, positive feedback)<br><br>*"The setup and installation of the device needs much more instructions from a patient standpoint … it's not really patient friendly … I'm not sure what the problem was. Could it have just been a glitch or whatever ... But it's great to be able to do it at home, if it works"* (US patient, challenge/barrier and positive feedback)<br><br>*"It is a concern, yes. When you think about the maintenance aspect, I think it would have to be clean. And if I were to not handle it properly, I think that something strange might happen. I think that it might a little bit of a hassle and I might not use it"* (Japanese patient, suggestion)<br><br>*I'm just worried about using it on my own, by myself … I would really wonder as to whether I'm using it properly. And just like I said earlier, there would be the maintenance, right? And I'm really worried about that, too"* (Japanese patient, suggestion and challenge/barrier)	*"So, I'm there to be a coach, and I don't think ultimately – I'm not a PFT tech, but my being present certainly didn't add any value. So, either you do without a coach or you have a better coach"* (US HCP, challenge/barrier)<br><br>*"Miss all those three attempts [home spirometry] and – three unsuccessful, so that's pretty bad … let's say it was a huge technological problem with a tech savvy guy [referencing patient]"* (US HCP, challenge/barrier)<br><br>*"But with these new technologies, if there is a problem, if it is minor everything gets delayed. So that's why I insist that these apps should be very user friendly, very easy to handle, to use, because most of the patients with pulmonary fibrosis, are 70 or 80 year old patients"* (Spain HCP, challenge/barrier)<br><br>*"When I used it and I've explained it and I've had patients use it elsewhere. And a lot of the times they don't really know how to use the other affiliated devices, not the actual spirometry, they know how to do the spirometry"* (Japanese HCP, challenge/barrier and positive feedback)
Blood tests	*"There's a lot of blood tests ... And when I saw this, I thought, wow, that's a lot. And each time ... each time you get blood tests right for example"* (Japanese patient, challenge/barrier)<br><br>*"I was wondering about that and the blood test frequency, a little bit high I would say"* (Japanese patient, challenge/barrier)	-
Patient reported outcomes incl. quality of life	*"I suffer from chronic fatigue … Questionnaires are taken at every visit so that's going to be a 30-minute function so I'm not sure that, that needs to be done frankly [discussing burden of visits]"* (US patient, challenge/barrier)<br><br>*"The questionnaires can be done from home. My issue here is new technologies like I said but I guess you would be helping me"* (Spanish patient, suggestion)<br><br>*"Now for the pulmonologist, the breathing is that big target because it's a measurable thing, but for me the improvement, the quality of life is, can I walk upstairs better, can I perform things better, can I take a shower and put on clothes at the same time within the same hour? Those are things that are important to me"* (US patient, suggestion)	*"I think if they trusted me then I think that I could tell them about this [discussing patient reported outcomes including suicidality] but this is the first time that I was … meeting them and to be explaining such was a little bit scary"* (Japan HCP, challenge/barrier)

Caregiver assessments	*"Sometimes the caregiver really knows … living with someone, I think you have a better perspective. And I think it'd be valuable for you to get the caregiver's opinion … because they're with them all the time"* (US caregiver, suggestion)	-
**Trial visit schedule and duration**		
Trial length	*"The total the 52 weeks didn't seem like a deal breaker but the greater the number I think the more people would find more onerous"* (US patient, challenge/barrier)	*"Some [patients] are going to drop off [discussing 52+ week trial]. Hopefully, you screen well at the beginning that you're going to get those motivated people"* (US HCP, challenge/barrier)
Visit frequency (incl. >52 weeks) and length	*"There's 10 plus visits in a year … then at each visit, it's at least a two-hour visit … that's a fair amount of hard work as a patient. And I'm just not sure that I'd be up to that amount of work … I probably would still go forward with it … but having looked at what my requirements are for it, it's just dampened my enthusiasm of it. I'm still going forward with it, I'm just not as excited"* (US patient, challenge/barrier)	*"Well, I think the longer the interval, the easier it is on patients. But when patients take a medication there may be fluctuations in the way they feel on a daily basis .. so that's why I think that every 12 weeks, for example, or every eight weeks will be good. That would be good. The way it is set up right now"* (Japanese HCP, positive feedback)
Visit length	*"I think two hours is reasonable"* (Japanese patient, positive feedback)<br><br>*"I think two hours would be probably the outer limit of length. I could do it, but I just think anything more than that is pushing it ... you're going to be dealing with people who aren't always having a good day"* (US patient, challenge/barrier)<br><br>*"Chronic fatigue syndrome; I only have five to six hours a day of energy to complete activities … so for me … the day is shot for me. And chronic fatigue is common amongst ILD patients, so we got to think about that"* (US patient, challenge/barrier)	*"I think that both of them [discussing simulation patients] did not think that two hours was that long ... The first patient seemed to think that that two hours was a little bit long. When you go for a physical … I think two hours is how much it takes and it's not really that bad for healthy individuals … but if people are in discomfort, two hours is really difficult, or it's hard on them so that's why I think two hours for those types of patients might be a little bit long"* (Japanese HCP, challenge/barrier)
Visit flexibility	*"As long as they're I would say probably morning to early afternoon [discussing visits] is better for most of the patients. His body starts shutting down probably by three in the afternoon. He starts going downhill"* (US caregiver, suggestion)<br><br>*"It would help that in working with the study coordinator, that they would have a little bit of flexibility. And if I would have a vacation planned and visit 6 that we could, we can move it out a week, but still be within that time period, and do something. So, we just have to see*" (US patient, suggestion)<br><br>*"So for instance, I don't know to change my time or the day of my visit, but as I say, I will do my best. I will do my best to stick to the visits that they will schedule for me"* (Spanish patient, suggestion)	-
Remote visit option	*"I would do whatever I needed to do, but I just thought it was much more reasonable than having to travel far, and the fact that you can do some things on the computer. I liked that"* (US caregiver, suggestion and positive feedback)<br><br>*"Yes, I would benefit indeed [from virtual visit option] because not all the days*	*"Remote consultations within the clinical trial for some of the test visits is very,*

	are the same" (Spanish patient, positive feedback)<br><br>*"I guess the most positive thing is that it can be done from home"* (Spanish patient, positive feedback)<br><br>*"I prefer visits. Well, I prefer … the person I care for to undergo tests for better control, for better monitoring … But if visits were to be virtual, my scoring will not change"* (Spanish caregiver, suggestion)<br><br>*"Because everything that has to be done through the computer is more difficult for me, so I'd rather go to a place in person and get the tests done. I come from a different generation"* (Spanish patient, challenge/barrier)	*very interesting. Because patients need to follow up with many visits, they need to travel to study center to the hospital wherever. Everyone benefits, everyone wins. The patients benefit … It is also a win for doctors because the doctors can … devote more time to the patients when they call them through video call … The future moves along those lines, I … remote consultations in clinical trials, I find it very much of a novelty highly innovative…"* (Spanish HCP, positive feedback)<br><br>*"I think that it could [motivate participants]. They are really suffering, right? … So I think that moving about and taking transportation is probably a little bit difficult for those individuals, moving about, that is. So that's why, if it would have remotely, then that would really be a strong thing from their perspective. Very, very good from their perspective"* (Japanese HCP, positive feedback)
**Participant support**		
Visit guide and reminders	*"I'm big on reminder emails … remind them of visits and remind them at this visit here's what we're going to do … just to set the expectation that it's going to take a while. And then … if there's anything that needs to be done beforehand. If you tell them up front … maybe that's a little bit helpful"* (US patient, suggestion)<br><br>*"If we had an agenda. If we had a schedule, I would feel less anguish. I would feel more safe"* (Spanish patient, suggestion)	-
Site accessibility	*"If you have a big site … you may have to walk a mile … I can't do a mile … I don't typically have a wheelchair, but I might need one for that … it'd be nice to get a shuttle from the parking lot, or get dropped off at a door, or something to know where I'm going and how I could best get there"* (US patient, suggestion)<br><br>*"Getting to the weekly visits, that can be difficult. My wife is in a wheelchair"* (US caregiver, challenge)<br><br>*"Either bus passes, or city transit, or parking if the study is done at a university that university credits, or the city passes, or either you were Uber credits or something along that line is good, because it's hard for some people to get around"* (US patient, suggestion)	-
Caregiver reimburseme nt/ compensation	*"At least in studies that I've seen, I've never seen study partner caregiver compensation. I highly encourage it because they have costs, and they have skin in the game"* (US patient, suggestion)	-
Childcare	*"[Discussing childcare] Not for me, but for other people, I think that's very important"* (US patient, suggestion)	

Trial navigators, mentors, and peer support	*"Well when you have a peer group you can feel more supported. Psychologically it's really helpful at least for me as a caretaker and for my mom as a patient to be able to share her experience with someone who's going through the same thing, I think that's very helpful"* (Spanish caregiver, suggestion)<br><br>*"It's a really good to have that person [discussing study care navigator] that you know you can always get a hold of because sometimes the doctor … is not available. So having that contact there that is always available … I think to navigate everything and again takes some of that stress level off of you"* (US caregiver, suggestion)<br><br>*"I think it's all about mental health and I'm thinking about maybe a psychologist, psychological support, having some visits with a professional where she can really explain or share those concerns. I think it goes around the same idea of the peer group or being linked to this trial beyond just a medical fact, the fact that it's personally and psychologically giving her something"* (Spanish caregiver, suggestion)	*"Respiratory patients … the more advanced their disease, the higher the anxiety they have. And that anxiety translates into they want results soon, they want brevity. So if we are offering a patient to take part in a clinical trial in the long term, the patient in return needs to find a reward or something positive, something that makes it worthwhile to go ahead with the trial … if he can see that the follow up is very close, the visits are regular, that he is, all his issues are taken care of, are being handled. And he can see that he is well monitored, well controlled, well supervised, that also facilitates adherence ... So patient wants to see outcomes, results and to be taken care of"* (Spanish HCP, suggestion)<br><br>*"Someone who listens to, someone who resonates, who sympathizes with you is very important from the patient's perspective – very important. They value that a lot. They really value someone who listens to them ... So that's why over 52 weeks then, if research over 52 weeks then that's going to be even more important"* (Japanese HCP, suggestion)
Information for primary care physician	*"Well, I guess that my primary care physician would like to hear about the … regular testing that I will be undergoing throughout the clinical trial … he refers me to other doctors … therefore it'll be very important for him to know about those tests, the outcomes, the results, how those tests were done"* (Spanish patient, suggestion)<br><br>*"More than the primary care, I think the pulmonologist would be the one who should be informed. The specialist … I think I would be telling him that I'm getting a trial to see what his opinion is. Once I'm in, I would like for him to have the tests and results at his disposal"* (Spanish patient, suggestion)<br><br>*"Maybe test results, anything to do with the test results in the trial … if the results could be sent to my PCP and be seen by my PCP as well, that would be great"* (Japanese patient, suggestion)	*"It really depends on whether the PCP is actually understanding of the patient's condition. For example, in terms of specific numbers and if that is explained to the patient verbally, I would have to say that things would differ so maybe test results could be used so if the doctor were to explain things properly to the patient in terms of the test results that would be really good I think"* (Japanese HCP, suggestion)<br><br>*"Patients with pulmonary fibrosis … do not go to the primary care physician, they do not go there for their respiratory assessments. That relationship is with the pulmonologist … if the patient needs adjustment due to worsening, I think that the treating pulmonologist should be aware of that. So the more information is given to the treating pulmonologist, the better"* (Spanish HCP, suggestion)
